# Methanolic Leaves Extract of *Ziziphus spina-christi* Inhibits Cell Proliferation and Migration of HER2-Positive Breast Cancer via p38 MAPK Signaling Pathway

**DOI:** 10.3390/ijms26020654

**Published:** 2025-01-14

**Authors:** Sumayyah Saeed, Arij Fouzat Hassan, Azza Suliman, Ala-Eddin Al Moustafa, Feras Alali

**Affiliations:** 1 College of Pharmacy, QU Health, Qatar University, Doha 2713, Qatar; ss2000190@qu.edu.qa (S.S.); ah1304418@qu.edu.qa (A.F.H.); aa1513782@qu.edu.qa (A.S.); 2 College of Medicine, QU Health, Qatar University, Doha 2713, Qatar; 3 Oncology Department, McGill University, Montreal, QC H4A 3T2, Canada

**Keywords:** *Ziziphus spina-christi*, HER2-positive breast cancer, anti-cancer, rutin, quercetin

## Abstract

Human epidermal growth factor receptor 2 (HER2) is a subtype of breast cancer that is associated with poor prognosis and low survival rates. The discovery of novel anti-cancer agents to manage this subtype of cancer is still needed. *Ziziphus spina-christi* (*ZSC)* is a plant species that is native to Qatar. It exerts various biological activities, including cytotoxicity as it contains different essential bioactive constituents, mainly rutin and quercetin. To examine the outcome of *ZSC* on HER2-positive breast cancer, we standardized the *ZSC* methanolic leaves extracted by Reverse Phase High-Performance Liquid Chromatography (RP-HPLC) analysis using the flavonoids rutin and quercetin as marker compounds. Here we used two HER2-positive breast cancer cell lines, ZR-75-1 and SK-BR-3, and the chorioallantoic membrane as an angiogenesis model. We found that *ZSC* extract significantly reduces viability, alters the normal morphological phenotype of HER2-positive breast cancer cells, and inhibits cell migration as well as colony formation; this is accompanied by deregulating different apoptotic markers such as Bax/Bcl-2 and NF-κB in both cell lines. Additionally, *ZSC* methanolic extract significantly represses the angiogenesis of the chorioallantoic membrane model. Moreover, the molecular pathway investigations pointed out that *ZSC* extract represses the activity of HER2 and p38 MAPK which could be the main pathways behind the effect of *ZSC* in HER2-positive cells. Collectively, our results support the potential role of *ZSC* in the management of HER2-positive breast cancer and form the basis for future investigations.

## 1. Introduction

Breast cancer is an aggressive and widely spread disease which accounts for around 12% of cancer cases worldwide [1]. Among women, it is considered the most prevalent cancer type and the major cause of death. In 2020, 2.3 million new incident cases have been reported [1,2]. The development of breast cancer occurs when normal breast cells that mainly originate from the interior epithelia of milk ducts or lobules mutate and multiply aggressively [3,4,5]. This will eventually lead to the growth of tumors in the breast. If not controlled, cancer cells develop and metastasize through the blood to other nearby tissues and lymph nodes [6]. Breast cancer is highly complicated and is therefore classified into four subtypes depending on the predominant cellular markers. Luminal A, luminal B, human epidermal growth factor receptor 2 (HER2) positive, and triple-negative breast cancer, which does not express any of these three receptors [3]. HER2-positive is an aggressive subtype of breast cancer comprising around 25% of total breast cancer cases. It is characterized by the increased expression of the HER2 receptor, which contributes substantially to the aggressiveness of breast cancer. This receptor belongs to the epidermal growth factor receptor family of tyrosine kinases which are responsible for cellular survival [7,8,9,10,11,12]. The activation of these receptors initiates tumorigenic signaling pathways resulting in several pro-survival processes that lead to the development and formation of tumors [3,10,13,14,15]. HER2-positive breast cancer is considered life-threatening with low survival rates, poor prognosis, high potential of metastasis, drug resistance, and considerable chance of recurrence [10]. Current treatment options for HER2-positive breast cancer are surgeries, chemo, hormonal, or targeted therapy. Targeted therapy toward the HER2 receptor is a crucial approach in the treatment of HER2-positive breast cancer. Several effective anti-HER2 agents have been developed in the past years, providing better outcomes and potentially enhancing survival [9,10,16,17,18,19]. However, they can cause serious side effects and acquire a high chance of developing drug resistance [20,21,22,23,24,25]. Despite the significant advancements in the diagnosis, treatment, and monitoring of HER2-positive breast cancer, it has not yet been conquered, and newly diagnosed cases are rising continuously. Hence, there is a need for further active investigations to explore novel therapeutic candidates that are effective, selective, and less harmful for the management of this subtype of breast cancer.

Medicinal plants play an essential role in the field of drug discovery and development, as they exhibit structural variability and are rich in bioactive molecules [26,27,28]. Potential drug candidates could be derived from medicinal plants and be used as leads for the innovation of novel agents for different therapeutic purposes [26,29,30]. Today it is well known that about 65% of approved anti-cancer drugs have been inspired by natural products [31]. *Ziziphus spina-christi* (*ZSC*) is a plant species that grows in tropical regions and belongs to the genus *Ziziphus*, which is classified under the Rhamnaceae family [32]. *ZSC* has been extensively utilized in traditional medicine for the treatment of different diseases [33]. Moreover, several investigations reported the potential use of *ZSC* in the treatment of several diseases including cancer [32,33]. In earlier studies, *ZSC* was screened for its phytoconstituents using different analytical techniques revealing its abundance with several groups of metabolites such as polyphenols, flavonoids, tannins, saponins, and alkaloids [33,34,35,36,37,38]; numerous flavonoids such as rutin, quercetin, catechin, apigenin, and naringenin had been identified, and quantified from different extracts of *ZSC* [33,35]. Indeed, phenolic compounds and flavonoids exhibit substantial anti-cancer properties, which support the anti-cancer potential of *ZSC* species [39,40]. Previously, *ZSC* anti-cancer effects have been examined in vitro against different cancer types such as breast, liver, cervical, colon, and lung [38,41,42,43,44,45,46]. In addition, previous in vivo studies have been conducted to prove its anti-cancer effects using different rat models with hepatocarcinoma and colon cancer [43,47]. However, its effect on HER2-positive breast cancer has not yet been investigated. Thus, we herein explored the effects of *ZSC* methanolic leaf extract on HER2-positive breast cancer cell lines ZR-75-1 and SK-BR-3. We also evaluated the anti-angiogenetic potential of *ZSC* extract using the chorioallantoic membrane of chicken embryos.

## 2. Results

### 2.1. Plant Extract Standardization Using Reversed Phase High-Performance Liquid Chromatography (RP-HPLC)

**Figure 1:**
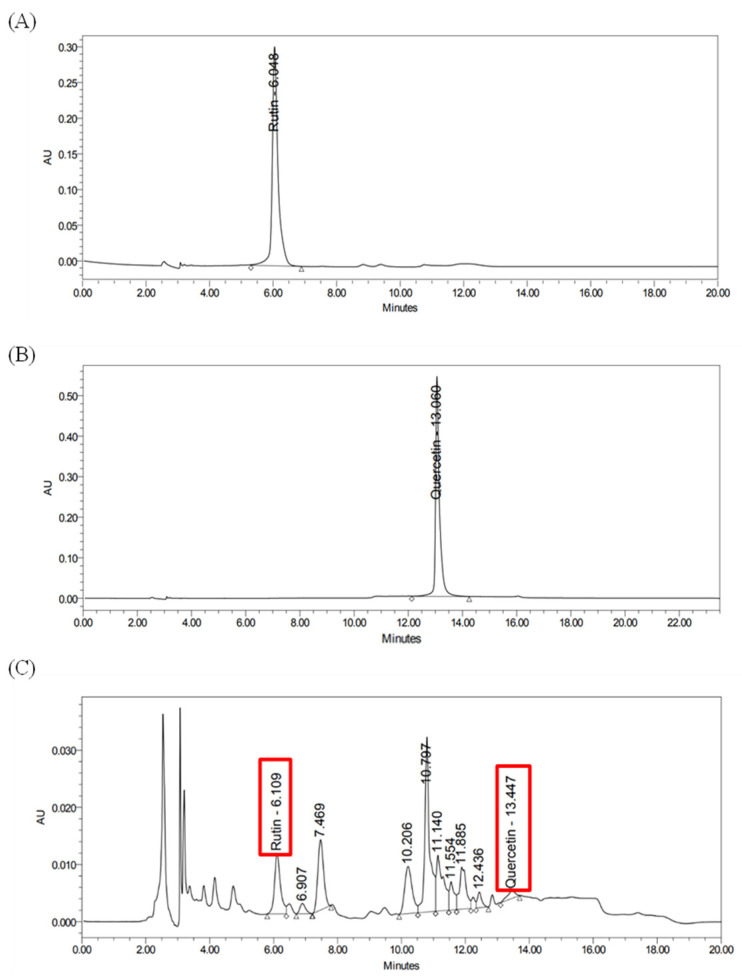
HPLC chromatograms of (**A**) pure rutin (**B**), pure quercetin, and (**C**) *Ziziphus spina-christi* (*ZSC*) methanolic leaves extract. Concentration: 0.5 mg/mL. Detection at 280 nm.

Based on the RP-HPLC standardization of the *ZSC* leaves extract, as described in the materials and methods section, using rutin and quercetin. The peaks related to both compounds were successfully detected in the resulting HPLC chromatogram of the established *ZSC* methanolic leaves extract at the same retention times as the reference peaks of the standard compounds as shown in Figure 1, in concentrations of 24 ± 1.9 µg/mL and 1.4 ± 0.2 µg/mL for rutin and quercetin, respectively.

### 2.2. Anti-Cancer Screening

#### 2.2.1. The Effect of *ZSC* on HER2-Positive Breast Cancer Cells

**Figure 2:**
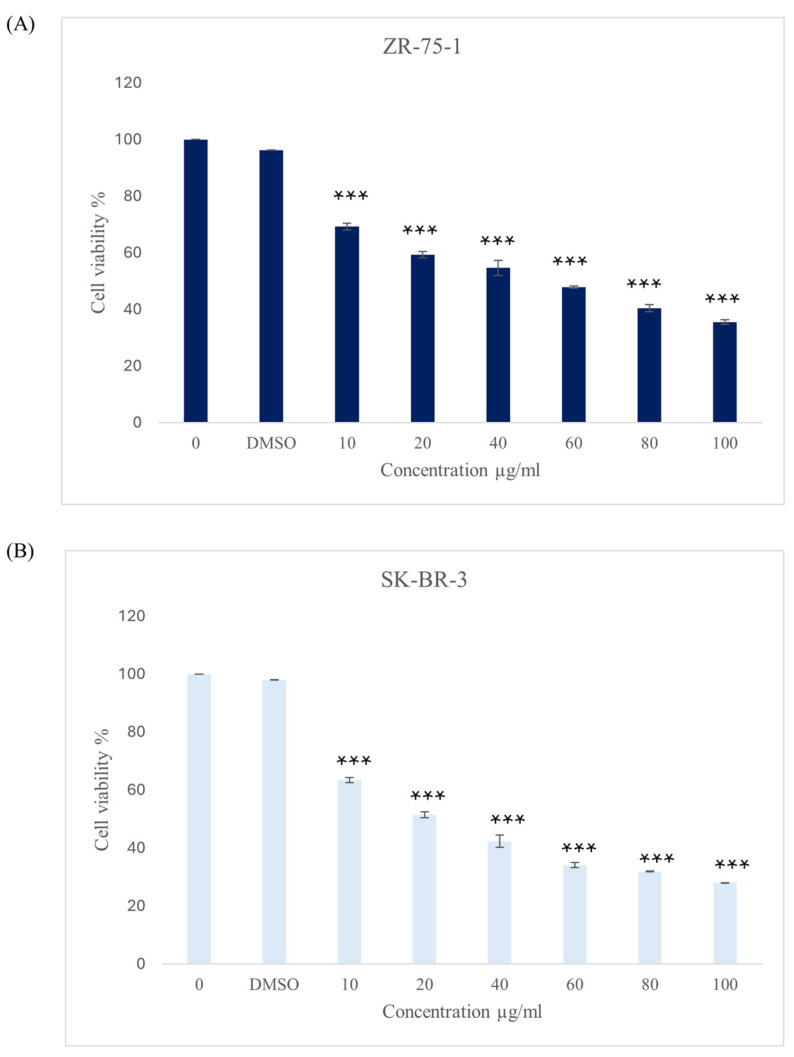
Effect of *Ziziphus spina-christi* (*ZSC*) extract on the viability of human epidermal growth factor receptor 2 (HER2) positive breast cancer cell lines (**A**) ZR-75-1 and (**B**) SK-BR-3 in relative with control after 48 h of incubation with a range of concentrations of the extract and dimethyl sulfoxide (DMSO) 0.5%. Data are expressed as mean values ± SEM, n = 3. One-way ANOVA test was conducted for statistical analysis followed by post hoc Tukey’s test to compare the groups and find the significance. Statistical significance was considered when the *p*-value was less than 0.05. *** *p* < 0.001.

**Figure 3:**
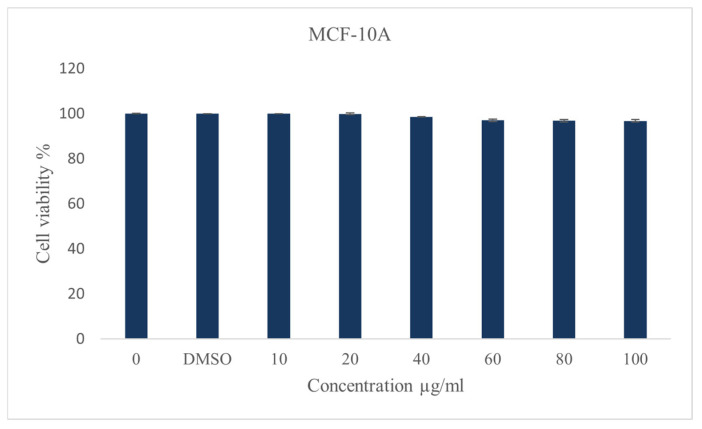
Effect of *Ziziphus spina-christi* (*ZSC*) extract on the viability of immortalized mammary epithelial cell line MCF-10A in relative with the control after 48 h of incubation with a range of concentrations of the extract. Data are expressed as mean values ± SEM, n = 3. One-way ANOVA test was conducted for statistical analysis followed by post hoc Tukey’s test to compare the groups and find the significance.

After 48 h of incubation of HER2-positive cell lines ZR-75-1 and SK-BR-3 with a range of concentrations of the methanolic leaves *ZSC* extract (10, 20, 40, 60, 80, and 100 µg/mL), we found that there is a significant reduction in cell viability in comparison with the untreated cells for both cell lines, as shown in Figure 2. The IC_50_ yielded 60 ± 0.3 µg/mL and 40.4 ± 0.38 µg/mL for ZR-75-1 and SK-BR-3, respectively. The cells were also treated with an equivalent concentration of dimethyl sulfoxide (DMSO), and it had no inhibitory effect on the cell viability of both cell lines. To evaluate the safety of *ZSC* extract and its selectivity toward cancer cells, the non-cancerous cell line MCF-10A was used as a control. The cells remained viable, and there was no significant decrease in the number of cells at the same extract concentrations used (Figure 3).

#### 2.2.2. The Effect of Rutin and Quercetin

**Figure 4:**
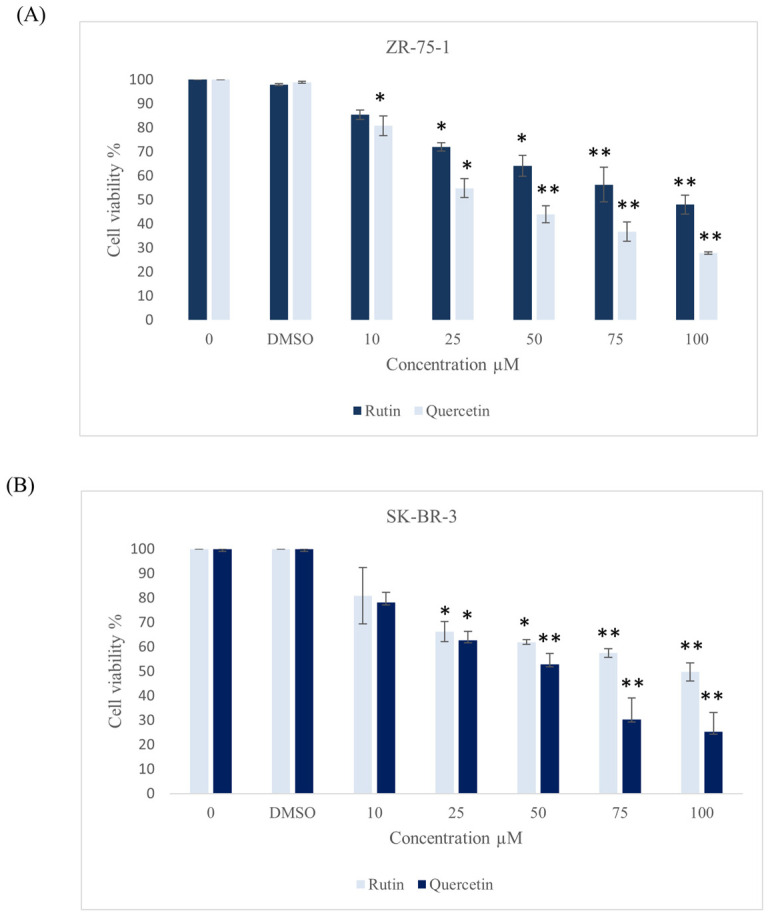
Effect rutin and quercetin on the viability of human epidermal growth factor receptor 2 (HER2) positive breast cancer cell lines (**A**) ZR-75-1 and (**B**) SK-BR-3 in relative with the control after 48 h of incubation with a range of concentrations of the compounds. Data are expressed as mean values ± SEM, n = 3. One-way ANOVA test was conducted for statistical analysis followed by post hoc Tukey’s test to compare the groups and find the significance. Statistical significance was considered when the *p*-value was less than 0.05. *p* < 0.05 *, *p* < 0.01 **.

**Figure 5:**
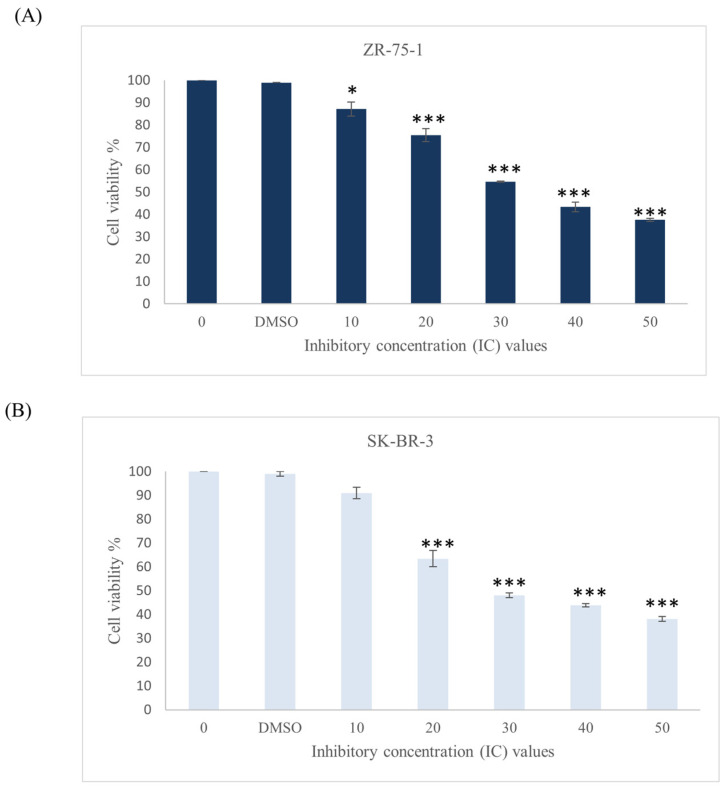
Effect of the combination of rutin and quercetin on the viability of human epidermal growth factor receptor 2 (HER2) positive breast cancer cell lines (**A**) ZR-75-1 and (**B**) SK-BR-3 in relative with the control after 48 h of incubation with a range of previously calculated inhibitory concentrations of the compounds. Data are expressed as mean values ± SEM, n = 3. One-way ANOVA test was conducted for statistical analysis followed by post hoc Tukey’s test to compare the groups and find the significance. Statistical significance was considered when the *p*-value was less than 0.05. * *p* < 0.05, *** *p* < 0.001.

Since rutin and quercetin are major components of ZSC, we examined the outcome of these two compounds on our two cell line models, ZR-75-1 and SK-BR-3. Different concentrations were used ranging from 10 to 100 µM for 48 h. The percentage of cell viability was significantly reduced in both cell lines (Figure 4). For rutin, the values were 85.66 ± 3.8 µM and 89.1 ± 1 µM for ZR-75-1 and SK-BR-3, respectively. Whereas for quercetin, the IC_50_ values were 55.19 ± 1 µM and 51.4 ± 2.7 µM for ZR-75-1 and SK-BR-3, respectively. This implies that quercetin exhibited a more potent cytotoxic effect than rutin in both cell lines. To evaluate the cytotoxic effect of both compounds in combination against HER2-positive breast cancer, ZR-75-1 and SK-BR-3 cell lines were treated with gradually increasing concentrations of each compound. After 48 h of treatment, a significant reduction in cell viability was observed in both cell lines starting from lower inhibitory concentrations (Figure 5). The IC_50_ values dropped from 85.66 ± 3.8 µM to 57.7 ± 0.7 µM and from 55.19 ± 1 µM to 35.9 ± 0.7 µM for rutin and quercetin, respectively, in ZR-75-1 cell line. Similarly, for the SK-BR-3 cell line, the values dropped from 89.1 ± 1 µM to 57.6 ± 0.015 µM and from 51.4 ± 2.7 µM to 30.9 ± 0.015 µM for rutin and quercetin, respectively.

#### 2.2.3. Morphological Examination

**Figure 6:**
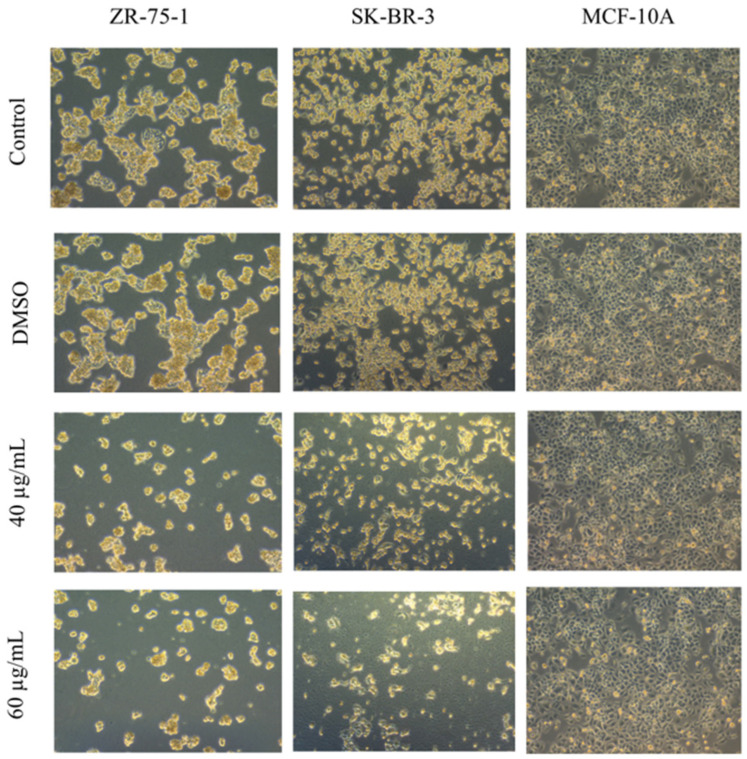
Cell morphology of human epidermal growth factor receptor 2 (HER2) positive breast cancer and immortalized mammary epithelial cell lines after 48 h of treatment with *Ziziphus spina-christi* (*ZSC*) extracts. The magnification scale of the images is 10×. n = 3.

In concordance with the results obtained from the cell viability experiment, there was a marked change in cellular morphology of ZR-75-1 and SK-BR-3 cell lines after 48 h of treatment with the IC_50_ values of *ZSC* extract on both cell lines (40 and 60 µg/mL). As shown in Figure 6, both cell lines appeared stressed, deformed, and shrunken. In addition, many cells were floating in the media with a noticeable reduction in the number and contact among cells in both cell lines. On the other hand, when MCF-10A cells were treated with *ZSC* extract (40 and 60 µg/mL) to evaluate their safety, the morphology of the cells was maintained. The cells were healthy and confluent, with no apparent changes or abnormal observations on their phenotype (Figure 6).

**Figure 7:**
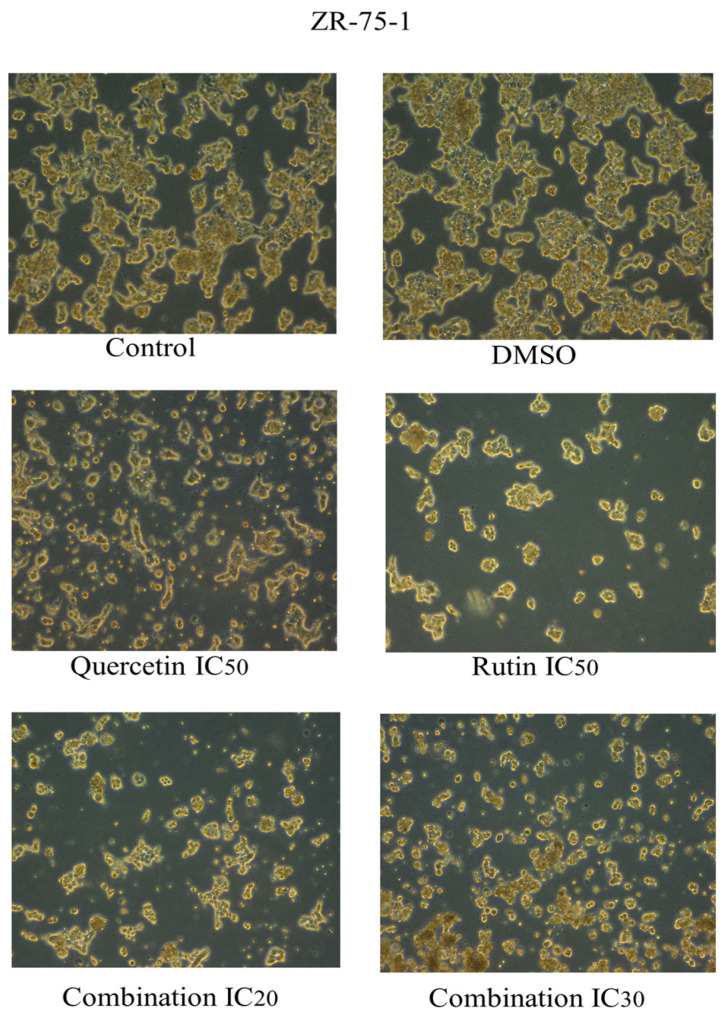
Cell morphology of human epidermal growth factor receptor 2 (HER2) positive breast cancer cell line ZR-75-1 after 48 h of treatment with rutin, quercetin, and rutin/quercetin combination at different inhibitory concentrations. The magnification scale of the images is 10×. n = 3.

**Figure 8:**
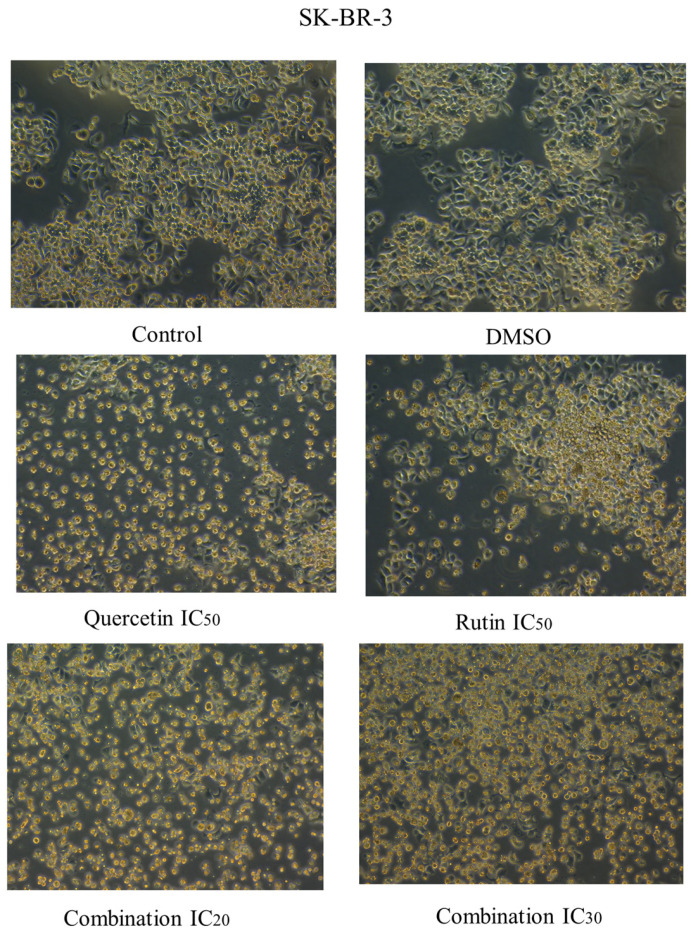
Cell morphology of human epidermal growth factor receptor 2 (HER2) positive breast cancer cell line SK-BR-3 after 48 h of treatment with rutin, quercetin, and rutin/quercetin combination at different inhibitory concentrations. The magnification scale of the images is 10×. n = 3.

Likewise, the same morphological changes were observed in the groups treated with the compounds rutin and quercetin, with more apparent effects observed in cells treated with their combinations (Figure 7 and Figure 8).

#### 2.2.4. Colony Formation

**Figure 9:**
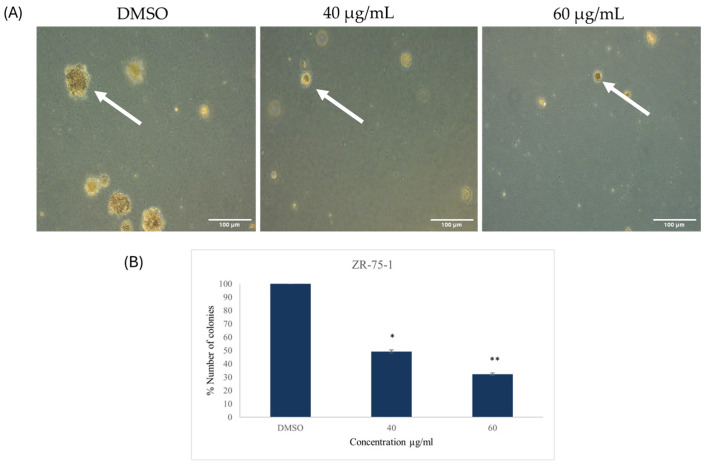
(**A**) Colony formation of human epidermal growth factor receptor 2 (HER2) positive breast cancer cell line ZR-75-1 after 21 days of treatment with 40 and 60 µg/mL of *Ziziphus spina-christi* (*ZSC*) extract. The magnification scale of the images is 10×. n = 3. (**B**) Number of the colonies of HER2-positive breast cancer cell line ZR-75-1 after 21 days of treatment with 40 and 60 µg/mL of *ZSC* extract relative to the dimethyl sulfoxide (DMSO) as negative control. The arrows in the figure point to the colonies. For statistical analysis, the One-way ANOVA test was conducted followed by post hoc Tukey’s test to compare the groups and find the significance. Statistical significance was considered when the *p*-value was less than 0.05. * *p* < 0.05, ** *p* < 0.01.

**Figure 10:**
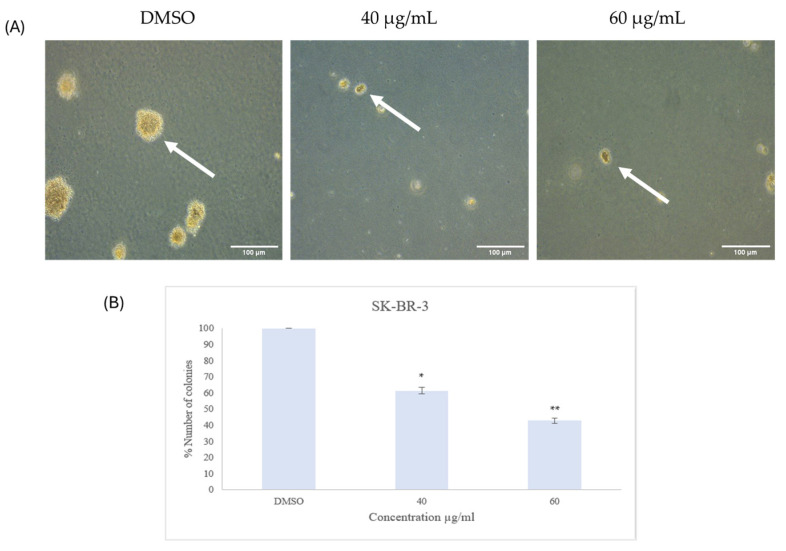
(**A**) Colony formation of human epidermal growth factor receptor 2 (HER2) positive breast cancer cell line SK-BR-3 after 21 days of treatment with 40 and 60 µg/mL of *Ziziphus spina-christi* (*ZSC*) extract. The magnification scale of the images is 10×. n = 3. (**B**) Number of the colonies of HER2-positive breast cancer cell line SK-BR-3 after 21 days of treatment with 40 and 60 µg/mL of *ZSC* extract relative to the dimethyl sulfoxide (DMSO) as negative control. The arrows in the figure point to the colonies. For statistical analysis, the One-way ANOVA test was conducted followed by post hoc Tukey’s test to compare the groups and find the significance. Statistical significance was considered when the *p*-value was less than 0.05. * *p* < 0.05, ** *p* < 0.01.

A soft agar colony formation assay was performed to observe the formation of colonies in soft agar, which reflects tumor formation in vivo. The observation of the cells throughout the incubation period revealed that at both concentrations, the extract had significantly decreased the number of colonies in both cell lines when compared with the control; in addition, upon visual observation, the sizes of the colonies formed in the treated groups were smaller than their respective controls (Figure 9 and Figure 10). After three weeks, the number of colonies in treated and untreated cells was quantified, showing 50.8 ± 1.2% and 67.7 ± 0.9% reduction at 40 and 60 µg/mL, respectively, in the ZR-75-1 cell line, whereas in SK-BR-3 cell line there was 38.6 ± 2.1% and 57.2 ± 1.8% reduction at 40 and 60 µg/mL, respectively (Figure 9 and Figure 10).

#### 2.2.5. Cell Migration

**Figure 11:**
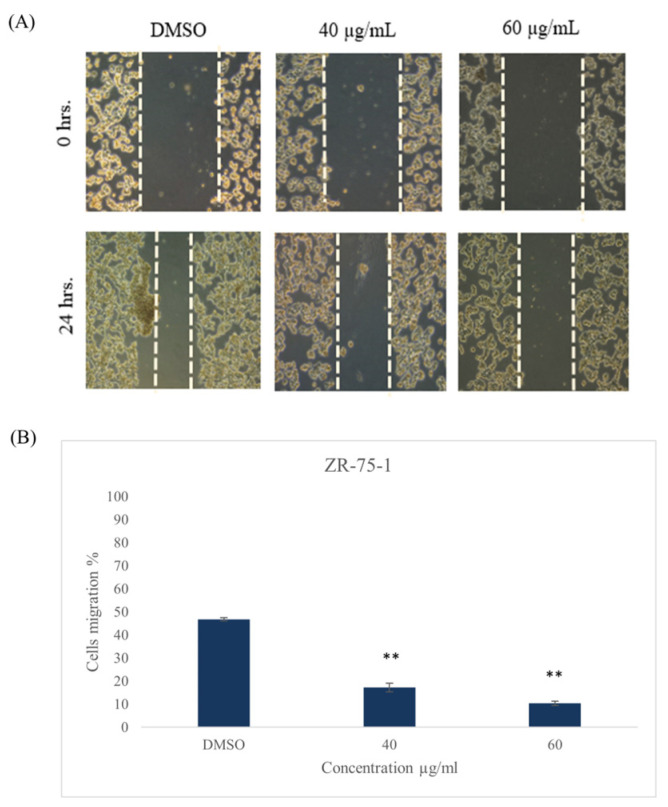
(**A**) Cell migration of ZR-75-1 cell line after 24 h of treatment with 40 and 60 µg/mL of *Ziziphus spina-christi* (*ZSC*) extract. The magnification scale of the images is 10×. (**B**) Quantitative analysis represents the percent of cell migration after 24 h. For statistical analysis, the one-way ANOVA test was conducted followed by post hoc Tukey’s test to compare the groups and find the significance. Statistical significance was considered when the *p*-value was less than 0.05. ** *p* < 0.01.

**Figure 12:**
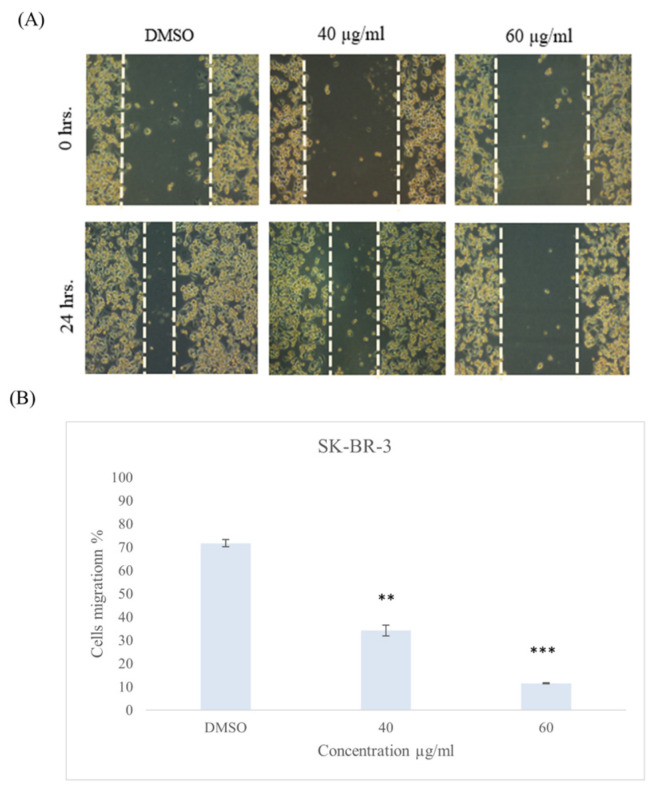
(**A**) Cell migration of SK-BR-3 cell line after 24 h of treatment with 40 and 60 µg/mL of *Ziziphus spina-christi* (*ZSC*) extract. The magnification scale of the images is 10×. (**B**) Quantitative analysis represents the percent of cell migration after 24 h. For statistical analysis, the one-way ANOVA test was conducted followed by post hoc Tukey’s test to compare the groups and find the significance. Statistical significance was considered when the *p*-value was less than 0.05. ** *p* < 0.01, *** *p* < 0.001.

After performing the scratch and treating the cells with 40 and 60 µg/mL of *ZSC* extract for 24 h, the results revealed the inhibitory potential of the extract on cell migration. The percentage of cell migration in the ZR-75-1 cell line was reduced to 17.2 ± 1.86% and 10.3 ± 0.9% at 40 and 60 µg/mL, respectively (Figure 11). In the SK-BR-3 cell line, the migration percentage was reduced to 34.2 ± 2.2% and 11.5 ± 0.9% at 40 and 60 µg/mL, respectively (Figure 12).

#### 2.2.6. Gene Expression and Molecular Pathway Study

**Figure 13:**
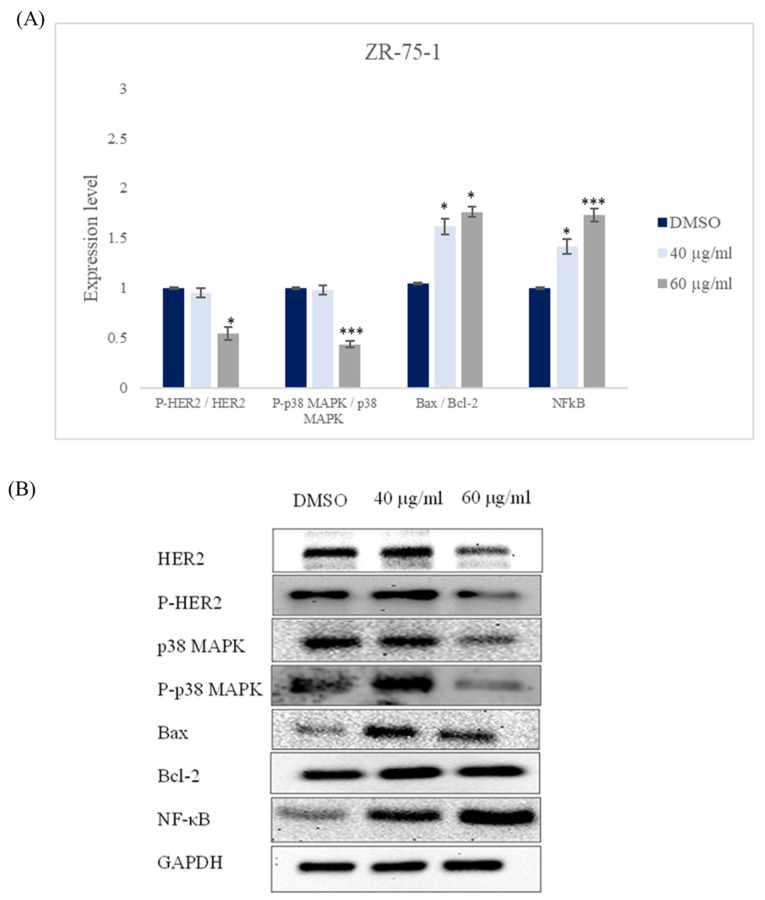
(**A**) Quantitative analysis of protein expression patterns in human epidermal growth factor receptor 2 (HER2) positive breast cancer cell line ZR-75-1 after 48 h of treatment with 40 and 60 µg/mL of *Ziziphus spina-christi* (*ZSC*) extract in relative with dimethyl sulfoxide (DMSO) as negative control. Values were normalized according to the housekeeping protein GAPDH. For statistical analysis, the one-way ANOVA test was conducted followed by post hoc Tukey’s test to compare the groups and find the significance. Statistical significance was considered when the *p*-value was less than 0.05. * *p* < 0.05, *** *p* < 0.001. (**B**) Representative western blot bands.

**Figure 14:**
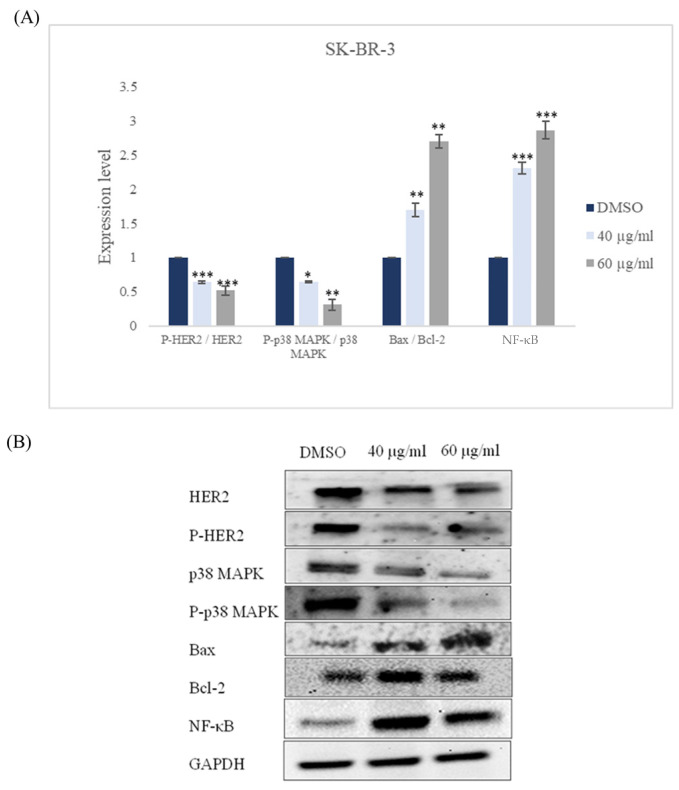
(**A**) Quantitative analysis of protein expression patterns in human epidermal growth factor receptor 2 (HER2) positive breast cancer cell line SK-BR-3 after 48 h of treatment with 40 and 60 µg/mL of *Ziziphus spina-christi* (*ZSC*) extract in relative with dimethyl sulfoxide (DMSO) as negative control. Values were normalized according to the housekeeping protein GAPDH. For statistical analysis, the one-way ANOVA test was conducted followed by post hoc Tukey’s test to compare the groups and find the significance. Statistical significance was considered when the *p*-value was less than 0.05. * *p* < 0.05, ** *p* < 0.01, *** *p* < 0.001. (**B**) Representative western blot bands.

Our data revealed that incubating ZR-75-1 and SK-BR-3 cell lines for 48 h with *ZSC* extract caused a significant increase in the Bax/Bcl-2 ratio in both cell lines which confirms apoptosis induction; this is accompanied by an up-regulation of NF-κB in our cell models (Figure 13 and Figure 14). Vis-a vis the molecular pathways, our data pointed out that *ZSC* extract provokes a downregulation in the expression patterns of HER2 and p38 MAPK which could be the main pathways behind the effect of *ZSC* in HER2-positive cell lines (Figure 13 and Figure 14).

### 2.3. Angigenesis Study

**Figure 15:**
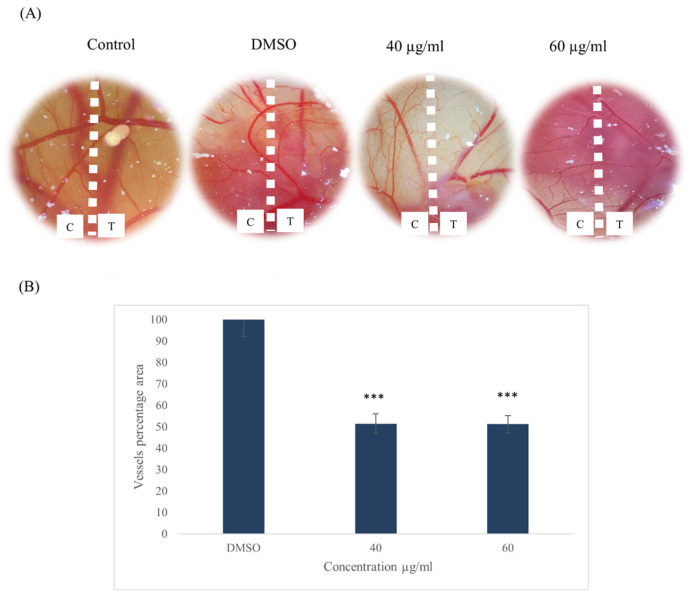
(**A**) Images representing the angiogenesis inhibition in the chorioallantoic membrane of chicken embryos after 48 h of incubation with 40, and 60 µg/mL of *Ziziphus spina-christi* (*ZSC*) extract. T: treated, C: control (not treated). (**B**) Quantitative analysis by AngioTool software version 0.6 showing the vessels’ percentage area. For statistical analysis, the one-way ANOVA test was conducted followed by post hoc Tukey’s test to compare the groups and find the significance. Statistical significance was considered when the *p*-value was less than 0.05. *** *p* < 0.001.

The embryos were treated with *ZSC* extract (40 and 60 µg/mL), DMSO (0.1%), and control for 48 h, then the angiogenesis bed for each embryo was visualized under the microscope. There was an apparent inhibition in the formation of blood vessels in the embryos exposed to the extract. After normalization with the unexposed area within the same embryo and comparison with the external controls, the results revealed that the extract had significantly reduced the formation of blood vessels, thus indicating its anti-angiogenetic potential. We found that *ZSC* extract inhibited the formation of blood vessels to 51.4 ± 4.4% and 51.2 ± 4% at 40 µg/mL and 60 µg/mL, respectively (Figure 15). The effect was dose-independent, as the percentage reduction was the same in each concentration, pointing out that 40 µg/mL was the least effective concentration for inhibiting angiogenesis.

## 3. Discussion

It is well-known that the HER2-positive breast cancer subtype is an aggressive disease with limited options for treatments. Thus, there is a need for new and effective therapeutic candidates to manage this subtype of cancer. Accordingly, in this study, we examined the outcome of *ZSC* extract on two human HER2-positive breast cancer cell lines. Our data revealed that the methanolic leaf extract of *ZSC* produced substantial effects against the two cell line models, ZR-75-1 and SK-BR-3. We herein report that there is a significant reduction in cell viability after treatment in both cell lines, which is consistent with previous studies that showed the cytotoxic effect of *ZSC* extracts against other subtypes of breast cancer, such as estrogen-positive and triple-negative [38,41,42]. As shown previously, *ZSC* significantly reduced the viability of MCF-7 when the cells were treated with a range of concentrations of the leaf extract, resulting in an IC_50_ value of 20 µg/mL [41,42]. In addition, different fractions of *ZSC* extract were examined against triple-negative breast cancer cell line MAD-MB-468 demonstrating a significant reduction in cell viability in a dose-dependent manner and resulting in an IC_50_ of more than 100 µg/mL for the most active fraction [38]. Similarly, another triple-negative breast cancer cell line, MDA-MB-231, responded with a reduction in cell viability after treatment with the ethanolic extract of *ZSC* [44]. Accordingly, it is evident that the potency of *ZSC* extract depends on the subtype of breast cancer. The extract exerted the most potent effect on MCF-7 (IC_50_ value: 20 µg/mL), followed by HER2-positive breast cancer cell lines ZR-75-1 and SK-BR-3 (IC_50_ values: 60 and 40 µg/mL, respectively), then triple-negative breast cancer cell lines MDA-MB-468 and MDA-MB-231 (IC_50_ value: ˃100 µg/mL). This could be due to the genetic variations of each cell line, which can affect the sensitivity of the cells toward the treatments. Collectively, these results confirm the antiproliferative effect of *ZSC* against different subtypes of breast cancer.

Additionally, morphological examination of our cell models after treatment supported the data obtained through cell viability by demonstrating clear signs of cell death. Gene expression study by western blot analysis revealed that the extract triggered apoptotic pathways via key apoptotic gene regulators, such as *Bax* and *Bcl-2*. There was a significant upregulation in the expression of the pro-apoptotic marker Bax, with no significant changes in the expression pattern of the anti-apoptotic marker Bcl-2 in both cell lines. This significant increase in the expression of Bax over Bcl-2 consequently increases the Bax/Bcl-2 ratio, which favors apoptosis. Our findings are concordant with previous studies on different cancer types [41,42,43]. Ghaffari et al. reported that *ZSC* leaf extract induces apoptosis in the MCF-7 cell line by activating the pro-apoptotic marker Bax [41]. Similarly, marked stimulation of apoptosis in the MCF-7 cell line was achieved using ethanolic *ZSC* extract and annexin V staining [42]. Furthermore, an in vivo study revealed that after injecting a hepatocellular carcinoma rat model with *ZSC* leaf extract, a significant downregulation of the anti-apoptotic protein Bcl-2 was observed, which enhanced apoptosis and tumor shrinkage [43].

Notably, *ZSC* is considered rich in secondary metabolites and bioactive groups such as flavonoids, phenols, alkaloids, saponins, and tannins [32,33]. Among them, rutin and quercetin are well-studied major bioactive constituents of *ZSC* with potential therapeutic benefits [33,34,35]. In our study, RP-HPLC analysis was conducted for standardization of the established *ZSC* methanolic leaf extract. The marker flavonoids rutin and quercetin were detected in the extract and quantified for their concentrations. They belong to the flavonoid group, a class of polyphenols that are highly abundant in medicinal plants and well known to possess prominent cytotoxic activities [39,40]. Multiple classes of flavonoids have been shown to exhibit significant cancer-preventative effects and anti-tumorigenic properties [48]. It was reported by several investigations that rutin and quercetin demonstrate considerable cytotoxic effects against different cancer types such as liver, cervical, ovarian, colon, prostate, lung, and estrogen receptor-positive breast cancers [49,50,51,52,53,54,55,56,57,58,59,60]. Quercetin has been reported to exert potent cytotoxicity against different cancer types, such as ovarian, endometrial, leukemia gastric, colorectal, and hepatic cancers, by blocking several tumorigenic events that enhance cancer survival and proliferation [61,62,63,64,65,66]. In addition, several evidence and investigations indicate quercetin’s effectiveness against breast cancer [40,53,55,56]. It triggers apoptosis in cancer cells and suppresses the generation of cancer stem cells, which are mainly involved in the recurrence of the disease [67]. Similarly, rutin possesses numerous biological activities, including anti-cancer, anti-inflammatory, and cardioprotective properties [58,68,69]. Rutin has been found to acquire substantial anti-cancer properties such as proliferation inhibition, apoptosis stimulation, cell cycle interruption, angiogenesis inhibition, and control of oxidative stress through different mechanisms and molecular pathways [54,57,70]. It had explicitly shown cytotoxic effects and the ability to overcome resistance toward chemo agents in breast cancer, thus reducing the chance of recurrence [53,57,71]. We herein performed an initial screening to test the cytotoxicity of both compounds against HER2-positive breast cancer cell lines ZR-75-1 and SK-BR-3. Our data revealed that rutin and quercetin exhibited prominent cytotoxic effects and significantly reduced cell viability of our two cell line models of HER2-positive breast cancer. Generally, quercetin exerted a more potent influence than rutin in both cell lines, as the resulting IC_50_ values were around 90 and 50 µM for rutin and quercetin, respectively. These data are consistent with the IC_50_ values reported in previous studies investigating the cytotoxicity of the compounds against other breast cancer cell lines [55,56,57]. In addition, morphological examination of both cell lines treated with the compounds confirmed their cytotoxicity indicating cell death and apoptosis. Thus, we believe that the effect of *ZSC* extract in the two HER2-positive breast cancer cell lines could be due to the presence of rutin and quercetin in addition to other active molecules in the extract.

Despite the observed cytotoxic effect of *ZSC* extract against HER2-positive breast cancer cell lines ZR-75-1 and SK-BR-3, there was no noticeable effect either on the viability or the morphology of non-cancerous cell line MCF-10A, which was used as a control for safety evaluation. The cells remained intact and healthy after exposure to a range of concentrations. This observation was consistent with previous studies testing the effect of *ZSC* extracts against different normal cell lines. In one study comparing the effects of the leaf extract on hepatocellular carcinoma cell line HepG2 and the normal melanocyte cell line HBF4, the results revealed a high selectivity index toward cancer cells and a considerably high IC_50_ value against normal melanocyte HBF4 cell line [43]. The same study further confirmed the safety of *ZSC* methanolic leaf extract in vivo by administering it to a healthy rat model with high doses, revealing that it did not affect the functions of the vital organs or induce death in this in vivo model [43]. Another study investigated the effect of *ZSC* extract against normal fibroblast cell line HFF-1, resulting in the survival of the cells with almost no reduction in viability [44].

Consequently, we investigated the *ZSC* extract on colony formation which showed that treatment of both cell lines with *ZSC* extract (40 and 60 µg/mL) resulted in a significant reduction in colony formation, reflecting the possibility of *ZSC* extract to suppress tumor development in vivo. Our findings are consistent with a previous in vivo study evaluating the development of the tumor in the colon cancer rat model, in which administering *ZSC* extract to the rats substantially reduced the development of precursor lesions (aberrant crypt foci) of colon tumors [47]. Further, a migration assay was conducted to evaluate the effect of *ZSC* extract on preventing the migration of ZR-75-1 and SK-BR-3 cells, thus reflecting its anti-metastatic potential. The results revealed that after treating the cells with the extract (40 and 60 µg/mL), there was a significant inhibition of cell migration in both cell lines.

Today, it is clear that several therapeutic strategies against HER2-positive breast cancer target the HER2 receptor [72]. We herein investigated the effect of *ZSC* extract on the expression pattern of HER2 receptors in ZR-75-1 and SK-BR-3 cell lines. The results showed a significant downregulation in the expression and the phosphorylation levels of the HER2 receptor in treated cells. On the other hand, one of the downstream kinases activated by the HER2 receptor is p38 MAPK, which activates fundamental tumorigenic events. Activation of p38 MAPK is associated with the progression and aggressiveness of HER2-positive breast cancer [73,74,75,76]. *ZSC* extract significantly suppressed the activation of the p38 MAPK receptor in both cell lines. Thus, our data pointed out that the effect of *ZSC* extract could be due to the suppression of the HER2/p38MAPK pathway in our cell line models.

Furthermore, our data revealed a significant upregulation of the transcriptional nuclear factor Kappa B (NF-κB) in ZR-75-1 and SK-BR-3 cell lines upon treatment with *ZSC* extract. Earlier studies reported that NF-κB plays an anti-tumorigenic role via the inhibition of the MAPK pathways [77,78,79,80]. Moreover, blockage of NF-κB resulted in anti-apoptotic action and induction of cell proliferation via ERK/MAPKs pathway in ovarian cancer cell lines [77]. This was supported by our results that revealed suppression of p38 MAPK and upregulation of NF-κB after exposure to *ZSC* extract. It is worth mentioning that although the role of NF-κB as an anti-apoptotic and pro-survival agent in cancer has been previously shown, several in vitro and in vivo investigations reported an anti-tumorigenic and pro-apoptotic potential of this gene via different mechanisms in other types of cancer such as skin, liver, and ovarian [76,81,82,83,84,85,86,87,88,89].

Finally, given the importance of angiogenesis on cancer progression especially in HER2-positive breast cancer, as the overexpression of HER-2 receptor is found to be highly associated with increased angiogenesis [90], we investigated the effect of *ZSC* extract on angiogenesis using the chorioallantoic membrane of the chicken embryo. Our angiogenesis study revealed the anti-angiogenetic effect of *ZSC* extract, which was evaluated for the first time, reflecting a substantial reduction in the vascularization of the embryos after exposure to 40 and 60 µg/mL of the *ZSC* extract.

## 4. Materials and Methods

### 4.1. Plant Collection and Extraction

The leaves of the *ZSC* tree were collected in July 2022 from the farm of Qatar University, Al-Khor, Qatar. The plant was taxonomically identified by the plant expert, Eng. Abu-Baker and a voucher specimen was kept at laboratories of the College of Pharmacy at Qatar University. The leaves were exposed to airflow at room temperature for three consecutive weeks to ensure complete dryness and water evaporation. The thoroughly dried leaves were ground by a mill until obtaining plant powder. The extraction process of *ZSC* leaves was performed by the maceration technique [91]. An aliquot of 1 g of the plant powder was placed in an Erlenmeyer flask and soaked in 50 mL absolute methanol (Sigma-Aldrich, St. Louis, MO, USA). The mixture was shaken, covered with parafilm, and kept overnight. The next day, the extract was collected and filtered through a glass funnel covered with a 0.20 μm pore size filter (Whatman, England, UK). The process was repeated for three consecutive days; each day after the collection of the solvent, a fresh volume of absolute methanol (50 mL) was added until the plant residue was entirely exhausted. After the three collection points, solvent evaporation was performed using a rotary evaporator (Butchi rotavapor R-215, Thermo Fisher Scientific, Waltham, MA, USA) and vacuum drying oven (Hi-Temp vacuum oven, Thermo Fisher Scientific, USA), then flushed with nitrogen to ensure complete evaporation of the solvent and pure extract powder.

### 4.2. Yield of Extraction

The extraction process yielded 32.5% of *ZSC* in the methanolic extract. An Aliquot of 10 mg of the extract powder was dissolved in 250 µL DMSO, providing a solution with a stock concentration of 40 mg/mL that was stored at 4 °C. For each in vitro assay, the extract’s stock solution was diluted in cell culture media to obtain a dimethyl sulfoxide (DMSO) percent less than 0.5%.

### 4.3. Plant Extract Standardization Using Reversed Phase High-Performance Liquid Chromatography (RP-HPLC)

RP-HPLC (Waters e2695, UV-PDA 2998 detector, Massachusetts, USA) was used for qualitative and quantitative detection of marker constituents of the extract. Waters© XBridge C18 (4.6 mm × 250 mm, five μm) column was used as a stationary phase. The mobile phase consisted of 0.5% acetic acid (Sigma-Aldrich) as solvent A and acetonitrile (Sigma-Aldrich,) as solvent B. The solvents were eluted in a gradient manner for 15 min per run as follows: 0–7 min (81.5% A); 7–9 min (65% A); and 9–15 min (82% A) with a flow rate of 1 mL/minutes. Before injecting the solvents into the system at each run, they were filtered with 0.20 μm pore size membrane nylon filters (Whatman) and degassed by bath sonication for 30 min using Branson Ultrasonics bath 5510. Each sample was dissolved in absolute methanol with a stock concentration of 0.5 mg/mL and filtered using a syringe filter with a pore size of 0.45 μm (Whatman). Then, 10 μL of the samples were injected into the column at room temperature. The peaks were detected at 280 nm by Waters© Empower chromatographic software version 3.8.0. An external calibration curve was constructed using standards to determine the exact concentrations of the marker compounds in the extract.

### 4.4. Cell Culture

HER2-positive breast cancer cell lines ZR-75-1 and SK-BR-3, in addition to MCF-10A, were obtained from American Type Culture Collection (ATCC, Rockville, MD, USA) and cultured in RPMI-1640 (Thermo Fisher Scientific) supplemented with 5% fetal bovine serum (PAN-Biotech, Aidenbach, Germany) and 1% penicillin-streptomycin 1X antibiotics (Thermo Fisher Scientific) to prevent bacterial growth. Cells were cultured in T-25 or T-75 flasks (Thermo Fisher Scientific) and then incubated at 37 °C and 5% CO_2_ until reaching 70% confluency for each experiment. Dimethyl sulfoxide (DMSO) (Thermo Fisher Scientific, USA) 0.5% was considered as a negative control in the experiments to exclude the effect of the vehicle.

### 4.5. Cell Viability Assay

ZR-75-1, SK-BR-3, and MCF-10A cell lines were cultured in supplemented RPMI-1640 media and seeded in 96-well plates (Thermo Fisher Scientific) at a 5000 cells/well density then incubated overnight. Afterward, fresh media containing different treatment groups was added to each well in a 100 µL/well volume. Treatment groups consisted of concentrations ranging from 10 to 100 µg/mL of *ZSC* extract, in addition to 10–100 µM of rutin (Adooq Bioscience, Irvine, AB, Canada), quercetin (Adooq Bioscience), and a 1:1 ratio of rutin/quercetin combination. After 48 h of incubation, 3-(4,5-Dimethylthiazol-2-yl)-2,5-Diphenyltetrazolium Bromide) 3-(4,5-dimethylthiazol-2-yl)-2,5-diphenyltetrazolium bromide (MTT) reagent (Thermo Fisher Scientific) was added to the plates and incubated for 4 h Later, DMSO was added to dissolve the formazan crystals. The absorbance was measured at a wavelength of 562 nm using a microplate reader (TECAN, Männedorf, Switzerland). The half-maximal inhibitory concentration (IC_50_) for the different treatments in each cell line was calculated using the linear equation of the resulting scatter plot.

### 4.6. Morphology Assay

ZR-75-1, SK-BR-3, and MCF-10A cell lines were seeded in 6-well plates (Thermo Fisher Scientific) in supplemented RPMI-1640 media at a density of 250,000 cells/well and then incubated overnight. Following, fresh media treated with the IC_50_ values of *ZSC* extract, rutin, quercetin, 1:1 ratio of rutin/quercetin combination, and DMSO 0.5% as negative control were added to the plates. After 48 h of incubation with the treatments, morphological changes were examined using a Leica SP8 UV/Visible inverted microscope (Leica Microsystems, Wetzlar, Germany) with a 10× objective lens. Pictures of the cells were captured using the Leica MC170 HD camera (Leica Microsystems) and Leica LAS EZ software version 3.4.0.

### 4.7. Colony Formation Assay

A soft agar colony formation assay was conducted as previously described by our group [92]. Briefly, a 2% stock solution of Noble agar (Sigma-Aldrich) was previously prepared in autoclaved deionized distilled water. In 6-well plates (Thermo Fisher Scientific), 1 mL of solidified agar layer was distributed in the wells. The first layer consisted of RPMI-1640 media solidified with 0.4% agar. The second layer consisted of ZR-75-1 and SK-BR-3 cells at a density of 5000 cells/well suspended in supplemented RPMI-1640 media that is treated with *ZSC* extract (40 and 60 µg/mL) and then solidified with 0.3% agar. The plates were incubated at 37 °C and 5% CO_2_ for 21 days. The size and number of colonies in each well were evaluated every two days using a Leica SP8 UV/Visible inverted microscope (Leica Microsystems) with a 10× objective lens. Images were taken each week using Leica MC170 HD camera (Leica Microsystems) and Leica LAS EZ software.

### 4.8. Migration Assay

ZR-75-1 and SK-BR-3 cells were seeded in 6-well plates in supplemented RPMI-1640 media at 300,000 cells/well density and incubated overnight. After 24 h, cells were incubated with serum-free RPMI-1640 media for four hours to achieve cell starvation. Afterward, a 200 µL pipette was used to scratch the middle of the cells’ monolayer and create a wound through which the cells could migrate. Any floating cells were removed by washing with 1X DPBS (Dulbecco’s phosphate buffer saline) (Thermo Fisher Scientific). Cells were later incubated for 24 h with fresh supplemented RPMI-1640 media treated with *ZSC* extract (40 and 60 µg/mL). Migration of the cells through the wound was observed using a Leica SP8 UV/Visible inverted microscope (Leica Microsystems) with a 10× objective lens. Images were taken using a Leica MC170 HD camera (Leica Microsystems) and Leica LAS EZ software. ImageJ software version 1.54d was used to quantify the area of wound closure.

### 4.9. Western Blot Analysis

ZR-75-1 and SK-BR-3 cells were cultured in supplemented RPMI-1640 media in Petri dishes (Thermo Fisher Scientific) at 2 million cells/dish density and then incubated overnight. After 24 h, the old media was replaced with fresh supplemented RPMI-1640 media that was treated with *ZSC* extract (40 and 60 µg/mL) and DMSO as a negative control, then cells were incubated for 48 h. Next, cell lysates were collected using cold SDS lysis buffer. The protein concentrations were determined using the Pierce BCA Protein Assay Kit (Thermo Scientific, Waltham, MA, USA). Subsequently, 30 µg of the protein samples were loaded on 10% polyacrylamide gels to run gel electrophoresis that separates the proteins based on their molecular weights using SDS polyacrylamide gel electrophoresis (SDS-PAGE)/Mini-PROTEAN Electrophoresis System (BioRad, Hercules, CA, USA). PageRuler™ Prestained Protein standard Ladder (10–250 kDa) (Thermo Scientific) was also loaded to indicate protein sizes. After separation, the proteins were transferred into 0.45 μm Polyvinylidene fluoride (PVDF) membranes by wet transfer process at 100 volts for 2 h; then 3% Bovine serum albumin (BSA) blocking buffer (Thermo Fisher Scientific) was added to the membranes before incubating them with the following primary antibodies: mouse anti-Bax (Thermo Scientific, ID# MA5-14003), mouse anti-Bcl-2 (Abcam, Cambridge, UK, ID# AB692), mouse anti-HER2 (Abcam, ID# AB16901), rabbit anti-P-HER2 (Abcam, ID# AB108371), rabbit anti-p38 MAPK (Cell Signaling Technology, Danvers, MA, USA, ID# 9212S), rabbit anti-P-p38 MAPK (Cell Signaling Technology, ID# 9211S), rabbit anti-NF-κB p56 (Abcam, ID# AB16502), and rabbit anti-GAPDH (Abcam, ID# AB9485). Next, membranes were probed with secondary antibodies, and the resulting protein bands on the blots were detected by ECL Western blotting substrate 46 (Thermo Fisher Scientific) using the Bio-Rad Chemidoc MP Imaging System. ImageJ software was used to quantify the intensity of the bands, which corresponds to the protein expression in the sample after normalization with the bands of the housekeeping protein GAPDH.

### 4.10. In Ovo Angiogenesis Assay

This assay was performed as previously illustrated by our group [93]. Briefly, fertilized White Leghorn chicken eggs (Arab Qatari for Poultry Production, Doha, Qatar) were incubated in a rotating MultiQuip Incubator with 60% humidity at 37 °C for five days. A small hole in the eggshell was opened to provide access to the chorioallantoic membrane. Then, 100 µL of DPBS 1X (Sigma-Aldrich) was added to the membrane to ease its removal. The embryos were treated with *ZSC* extract (40 and 60 µg/mL) and DMSO (0.1%). The treatments were loaded over coverslips (Sigma-Aldrich), which were placed over the embryos. The holes of the eggshells were then covered, and the embryos were incubated in the incubator. After 48 h, the angiogenesis bed of each embryo was examined using a Stemi 305 stereo microscope (Carl-Zeiss, Oberkochen, Germany). Images were captured to represent the areas exposed and unexposed to the treatments, which permits a more accurate analysis with minimal in-group variability. AngioTool software version 0.6 was used to analyze and quantify the percentages of vessel formation relative to the control.

### 4.11. Statistical Analysis

IBM^®^ SPSS^®^ software version 29.0.0.0 was used to perform statistical analysis. Through the program, a one-way analysis of variance (ANOVA) test was conducted to analyze the difference between the groups, followed by Tukey’s post-hoc test to specify the significance. Statistical significance was considered when the *p*-value was less than 0.05. The data were presented as mean ± standard error of the mean (SEM). Each experiment was conducted in three independent replicates (n = 3).

## 5. Conclusions

To the best of our knowledge, this is the first study investigating the cytotoxic potential of methanolic leaf extract of *ZSC* against HER2-positive breast cancer. Collectively, our findings revealed that the extract reduces cell viability and induces apoptosis in addition to suppressing important processes involved in the tumorigenesis and progression of the disease, such as migration, colony formation, and angiogenesis. Molecular pathway investigations revealed that the primary mechanism underlying the cytotoxic effect of the extract is through the deregulation and inactivation of the HER2/p38 MAPK signaling pathway. Additionally, our study demonstrates the effect of *ZSC* extract on the HER2 receptor that is majorly involved in the pathogenesis of the disease. Moreover, the *ZSC* extract was standardized using the marker compounds rutin and quercetin, which are flavonoids that exhibit anti-cancer effects. The compounds had proved their cytotoxicity against HER2-positive breast cancer as mono-agents and in combination. Finally, this study revealed that *ZSC* extract has a promising effect against HER2-positive breast cancer, therefore further pre-clinical investigations are necessary in order to evaluate its potential as a new therapeutic agent against HER2-positive breast cancer.

## Data Availability

The data presented in this study is available on request from the corresponding author.
